# Fertility Preservation for Child and Adolescent Cancer Patients in Asian Countries

**DOI:** 10.3389/fendo.2019.00655

**Published:** 2019-10-15

**Authors:** Seido Takae, Jung Ryeol Lee, Nalini Mahajan, Budi Wiweko, Nares Sukcharoen, Virgilio Novero, Antoinette Catherine Anazodo, Debra Gook, Chii-Ruey Tzeng, Alexander Kenneth Doo, Wen Li, Chau Thi Minh Le, Wen Di, Ri-Cheng Chian, Seok Hyun Kim, Nao Suzuki

**Affiliations:** ^1^Department of Obstetrics and Gynecology, St. Marianna University School of Medicine, Kawasaki, Japan; ^2^Department of Obstetrics and Gynecology, Seoul National University Bundang Hospital, Seoul, South Korea; ^3^Ferticity Fertility Clinics, New Delhi, India; ^4^Division of Reproductive Endocrionolgy and Infertility, Department of Obstetrics and Gynecology, Faculty of Medicine, Universitas Indonesia, Depok, Indonesia; ^5^Department of Obstetrics and Gynecology, Faculty of Medicine, Chulalongkorn University, Bankok, Thailand; ^6^St. Luke's Medical Center, Quezon City, Philippines; ^7^Section of Endocrinology and Infertility, Department of Obstetrics and Gynecology, University of Philippines Manila, Manila, Philippines; ^8^Kids Cancer Centre and Sydney Youth Cancer Service, Sydney Children's Hospital, Randwick, NSW, Australia; ^9^Nelune Comprehensive Cancer Centre, Prince of Wales Hospital, Randwick, NSW, Australia; ^10^School of Women's and Children's Health, University of New South Wales, Kensington, NSW, Australia; ^11^Melbourne IVF, Melbourne, VIC, Australia; ^12^Division of Infertility, Department of Obstetrics and Gynecology, Taipei Medical University Hospital, Taipei, Taiwan; ^13^Obstetrics and Gynecology, The Women's Clinic, Hong Kong, China; ^14^Reproductive Medicine Center, Second Military Medical University, Changzheng Hospital, Shanghai, China; ^15^Infertility Department, Tu Du Hospital, Ho Chi Minh, Vietnam; ^16^Department of Obstetrics and Gynecology, Ren Ji Hospital, School of Medicine, Shanghai Jiao Tong University, Shanghai, China; ^17^Center for Reproductive Medicine, Tenth People's Hospital of Tongji University, Shanghai, China; ^18^Department of Obstetrics and Gynecology, Seoul National University College of Medicine, Seoul, South Korea

**Keywords:** fertility preservation, child cancer patients, ovarian tissue cryopreservation, oncofertility, Asia

## Abstract

**Background:** At present, fertility is one of the main concerns of young cancer patients. Following this trend, “fertility preservation (FP)” has been established and has become a new field of reproductive medicine. However, FP for child and adolescent (C-A) cancer patients is still developing, even in advanced countries. The aim of the present study was to assess the barriers to FP for C-A patients by investigating the current status of FP for C-A patients in Asian countries, which just have started FP activities.

**Method:** A questionnaire survey of founding members of the Asian Society for Fertility Preservation (ASFP) was conducted in November 2018.

**Main findings:** Of the 14 countries, 11 country representatives replied to this survey. FP for C-A patients is still developing in Asian countries, even in Australia, Japan, and Korea, which have organizations or academic societies specialized for FP. In all countries that replied to the present survey, the patients can receive embryo cryopreservation (EC), oocyte cryopreservation (OC), and sperm cryopreservation (SC) as FP. Compared with ovarian tissue cryopreservation (OTC), testicular tissue cryopreservation (TTC) is an uncommon FP treatment because of its still extremely experimental status (7 of 11 countries provide it). Most Asian countries can provide FP for C-A patients in terms of medical technology, but most have factors inhibiting to promote FP for C-A patients, due to lack of sufficient experience and an established system promoting FP for C-A patients. “Don't know how to provide FP treatment for C-A” is a major barrier. Also, low recognition in society and among medical staff is still a particularly major issue. There is also a problem with cooperative frameworks with pediatric departments. To achieve high-quality FP for C-A patients, a multidisciplinary approach is vital, but, according to the present study, few paramedical staff can participate in FP for C-A patients in Asia. Only Australia and Korea provide FP information by video and specific resources.

**Conclusion:** The present study demonstrated the developing status of FP for C-A patients in Asian countries. More intensive consideration and discussion are needed to provide FP in Asian societies based on the local cultural and religious needs of patients.

## Introduction

Based on the Global Burden of Disease study, cancer incidence rate continues to increase in the world including Asian countries (1). Also, incidence of childhood cancer is increasing (2). Development of cancer therapy has resulted in increasing numbers of cancer survivors. In particular, more than 70% of child cancer patients will be cancer survivors (3). Unfortunately, one in 10 cancer patients experience fertility due to impairment in ovarian or testis function, as a result of the gonadotoxic treatments (chemotherapy and radiation therapy) as cancer therapy. Recently, several reviews of fertility preservation (FP) which based on assured clinical study have indicated the risk of infertility associated with specific diseases and therapies among different age groups. Especially, high-dose Alkylating agent represented by Cyclophosphamide may cause serious damage to gonads (4, 5). In addition, cancer itself and cancer treatment could cause the sexual dysfunction due to physical and psychological problems for cancer survivors (both men and women) including survivors of childhood cancer. To begin with, couple infertility and sexual dysfunction are highly prevalent in general population. Therefore, cancer and cancer treatment have possibilities getting worse this contemporary condition (6–8).

For adult patients with cancer, fertility preservation treatments have been established to improve quality of life for cancer survivors. In 2006, the “Oncofertility consortium” and “FertiPROTEKT,” which are representative associations to promote FP for young cancer patients, were established (9). The “International Society for Fertility Preservation (ISFP)” was established in 2009 as the first academic society specialized in FP treatments. Additionally, the “Japan Society for Fertility Preservation (JSFP)” and the “Fertility Preservation Society of India (FPSI),” and the “Asian Society for Fertility Preservation (ASFP)” were founded in 2012 and 2014, and 2015, respectively. Also, Australasian Oncofertility Consortium started 2015. As a consequence of efforts or actions to promote FP by these organizations, FP is now becoming a new field of reproductive medicine.

Based on the latest guideline that was updated by the American Society of Clinical Oncology (ASCO), only oocyte and embryo cryopreservation is endorsed as an “established method” for fertility preservation for female patients who face a threat to their own fertility due to cancer treatment (10). Meanwhile, ovarian tissue cryopreservation (OTC) is still an “experimental method” according to this guideline, although many experts believe that OTC fulfills the criteria for an “established method” (11, 12). The indications for OTC are specifically FP for child and adolescent patients and adult patients who do not have enough time to receive another fertility preservation treatment (5, 10, 11). Based on the literature, around 1,000 cases per year of oocyte cryopreservation (OC) for serious medical reasons and until now, more than 4,500 cases of OTC are performed in Europe (13), and more than 1,000 cases of OC and 200 cases of OTC are performed in Japan as FP (2006-2016, unpublished data). For male cancer patients, sperm cryopreservation before receiving chemotherapy is strongly recommended as the sole effective FP treatment. Hormonal therapy is not recommended as FP treatment for men. Testicular tissue cryopreservation (TTC) with later re-implantation is considered a highly experimental method (10). To determine the FP procedure for child and adolescent (C-A) patients, sexual maturity as we say “puberty” is one of important factors. It is menarche for female and spermarche for male. Generally, OC is the FP procedure which method is the most likely to result in subsequent pregnancy, but this is only for post-menarchal females (those who have begun to menstruate) since it would require developing follicles. Therefore, for pre-pubertal females, OTC is the only FP option. As a FP options for males, sperm cryopreservation is the most established option and should be offered to all peri- and post-pubertal male adolescents with a fertility-threatening situation. Although the age at which to offer sperm cryopreservation is unclear, an adequate semen specimen can be obtained in adolescents as young as 11 years of age. For pre-pubertal boys with lack of mature sperm, TTC is solely option as FP treatment (14, 15). At present, there are only two live birth cases from transplanted ovarian tissues that were cryopreserved before menarche, and there are no live birth cases from patients who underwent TTC (15, 16). In addition, there are few reports of OC for C-A patients. Even OC for late teenagers is still challenging because it needs ovarian stimulation with multiple hormonal injections and follicle monitoring using ultrasound, with subsequent oocyte retrieval under sedation or anesthesia (these procedures need a transvaginal approach) (15, 17). For these reasons, FP for C-A patients is still uncommon compared with FP for adult patients, even in advanced countries performing FP although they have OTC and TTC cases for infant (18, 19). The aim of the present study was to assess the barriers to FP for C-A patients by investigating the current status of FP for C-A patients in Asian countries whom are members of the Asian Society of Fertility Preservation (ASFP).

## Materials and Methods

### Survey Design

On November 2018, a survey was sent to country representatives of ASFP (Australia, China, Hong Kong, India, Indonesia, Japan, Korea, Philippines, Taiwan, Thailand, Vietnam, Pakistan, Singapore, Turkey) to collect information about the current status of FP services for child patients and the barriers that inhibit promoting this treatment. The participating countries gross national income per capita is very different (five high income countries, three upper-middle income countries, five lower income countries, and one with no data). The survey was approved by the institutional review board of our institution with revisions in keeping with the Declaration of Helsinki. The final version was sent by email to 14 contacts of the ASFP.

Potential survey participants were identified from existing members of the ASFP and international experts in the field. Potential participants received an email with an invitation to participate in the survey. Following the initial email, each participant received two reminders, one on November 1, 2018 and one on November 15, 2018, in order to maximize the number of responses.

### Survey Inclusion/Exclusion

Surveys were excluded from the analysis if participants failed to provide contact or identification information, if the survey was left blank, or if duplicate responses were submitted.

### Survey Questions

Survey participants were asked a total of 12 questions about the following areas: organization to promote FP treatment, patient access to medical professionals, current status of FP for adult and child patients, barriers that inhibit promotion of FP for C-A patients, and systems for providing information about FP for child patients. Three questions were dichotomous scaled questions (yes/no) with space for providing open-ended comments. Three questions were multiple-choice format, where only one answer could be selected. Four questions were multiple response questions, where participants could select one or more answers. One question was for free descriptive answer, and one was defining the priority order.

### Analysis of Survey Results

Survey responses were exported to Microsoft Excel. The dichotomous and multiple response questions were coded with numerical values to facilitate statistical analysis.

### Ethics Approval and Informed Consent

The present study was approved by the IRB of St. Marianna University (approval No. 4191, UMIN000035723). This survey is questionnaire survey targeted to medical professionals (representatives of society). On the explanation of this survey, we had written about consent to participate this survey at the front of questionnaires. We told them to reply when they could agree with participating this survey as participants.

## Results

### Organizations to Promote FP, Patient Access to Medical Professionals, and Current Status of FP for Adult Patients (Table 1)

From the 14 countries, 11 country representatives replied to the survey. Of the 11 countries, five had organizations or academic societies to promote FP, and three countries (Australia, Japan, and Korea) had organizations or academic societies that are specialized for FP in the true sense, whereas two (China and Indonesia) had a committee or branch society of a large academic society in the area of reproductive medicine or maternal-child health medicine. Two countries (Hong Kong and Philippines) are planning to establish organizations or academic societies specialized for FP. Although most countries do not have aid funds or insurance for FP, only Australia has a registration system for FP which requires individual patient consent and partial financial assistance or insurance system (Medicare) covering extensive FP treatment [embryo cryopreservation (EC), OC, consultation, ovarian transposition, sperm cryopreservation (SC)]. Also, Korea has partial funds for FP treatment (EC only).

**Table 1:** Organizations to promote FP, patient access to medical professionals, and current status of FP for adult patients in Asian countries.

		**Australia**	**China**	**Hong Kong**	**India**	**Indonesia**	**Japan**	**Korea**	**Philippines**	**Taiwan**	**Thailand**	**Vietnam**
Specialized organization for FP		Yes	Yes	No (in planning)	No	Yes	Yes	Yes	No (in planning)	No	No	No
Name of the organization		FUTuRE Fertility	Chinese Maternal and Child Health Association	(Hong Kong Society of Reproductive Medicine)	FPSI (Fertility Preservation Society of India)	Indonesian Association for IVF	JSFP (Japan Society for Fertility Preservation)	KSFP (Korea Society for Fertility Preservation)	PSFP (Philippine Society of Fertility Preservation)	–	–	–
Aid fund or insurance for FP		Yes (several)	No	No (in planning)	No	No	No (in planning)	Yes (EC only)	No	No	No	No
FP for female	EC	Yes (100–199)	Yes (>200)	Yes (1–49)	Yes (>200)	Yes (1–49)	Yes (100–199)	Yes (1–49)	Yes (6)	Yes (1–49)	Yes (1–49)	Yes (1–49)
	OC	Yes (100–199)	Yes (>200)	Yes (1–49)	Yes (>200)	Yes (1–49)	Yes (100–199)	Yes (1–49)	Yes (6)	Yes (1–49)	Yes (1–49)	Yes (1–49)
	OTC	Yes (10)	Yes (1–49)	No	Yes (3)	Yes (1–49)	Yes (38)	Yes (1–49)	Yes (1)	Yes (1–49)	Yes (1–49)	No
	GnRHa	Yes (unknown)	Yes (>200)	Yes (rare) (1–49)	Yes (>200)	Yes (1–49)	Yes (not standard)	Yes (1–49)	Yes (6)	Yes (1–49)	Yes (1–49)	No
FP for male	SC	Yes (>200)	Yes (1–49)	Yes (1–49)	Yes (>200)	Yes (1–49)	Yes^a^ (around 100)	Yes (1–49)	Yes (6)	Yes (1–49)	Yes (1–49)	Yes (1–49)
	TTC	Yes (1)	Yes (1–49)	No	Yes (>200)	Yes (1–49)	Yes (rare)	No	No	Yes (1–49)	No	No

*FP, fertility preservation; EC, embryo cryopreservation; OC, oocyte cryopreservation; OTC, ovarian tissue cryopreservation; GnRHa, gonadotropin releasing hormone agonist; SC, sperm cryopreservation; TTC, testicular tissue cryopreservation. ^a^Based on literature (49). Adult patients can be provided EC, OC, and SC as FP in all countries. Compared with OTC, TTC is an uncommon FP treatment. Only Australia and Korea provide funds for FP for patients*.

In all countries that replied to the survey, the patients can receive EC, OC, and SC as FP. Compared with OTC, TTC is uncommon FP treatment because of its still extremely experimental status. Therefore, even Australia, which is an advanced country for FP, has only one institution that has ethics approval for TTC although TESE can be done in post-pubertal patients in a number of centers if required.

### Current status of FP for C-A Patients (Table 2)

All of Asian countries have experience of FP for C-A patients. However, in most countries, the opportunities for FP for C-A patients are limited compared with FP for adult patients, because all participants (except for Indonesia) chose “not so often” regarding opportunities for FP for C-A patients. The main reasons were “not enough information for physicians, oncologists, patients and family” and “lack of public awareness.” Also, the numbers of facilities that can provide FP treatment for C-A patients are limited. Especially, in Australia, the facilities that can do OTC and TTC are strictly consolidated.

**Table 2:** Current status of FP for C-A patients in Asian countries.

			**Australia**	**China**	**Hong Kong**	**India**	**Indonesia**	**Japan**	**Korea**	**Philippines**	**Taiwan**	**Thailand**	**Vietnam**
Experience with FP for C-A patients			Not very often	Not very often	Not very often	Not very often	Most of the time	Some of the time	Some of the time	Not very often	Not very often	Not very often	Not very often
Reason or comments			Routinely only two centers done	Not enough information	Not enough information, lack of oncology support	Oncologist and parents are reluctant to provide FP	Two centers can provide FP	Not enough information, patient's disease	Lack of information to physicians, parents, patients	Fertility-sparing surgery and radiation shielding are done	Lack of public awareness	Parents concerned about cancer treatment more than FP	Lack of information, FP for C-A patients have not been established
FP for female	Children (0–14 y.o)	OC	No	No	No	Yes (>200)	Yes (1–49)	Yes (rare)	Yes (1–49)	No	Yes (1–49)	Yes (1)	No
		OTC	Yes (4)	Yes (1–49)	No	Yes (3)	Yes (1–49)	Yes (less than 38)	Yes (1–49)	Yes (1)	Yes (1–49)	Yes (1–49)	No
		GnRHa	No	Yes (>200)	Yes (1–49)	Yes (>200)	Yes (1–49)	Yes (not standard)	Yes (1–49)	No	Yes (1–49)	–^b^	No
	Adolescents (≥15 y.o)	OC	Yes (100–199)	Yes (1–49)	Yes (1–49)	Yes (>200)	Yes (1–49)	Yes (not so many)	Yes (1–49)	Yes (6)	Yes (1–49)	–^b^	Yes (1–49)
		OTC	Yes (10)	Yes (1–49)	No	Yes (3)	Yes (1–49)	Yes (less than 38)	Yes (1–49)	Yes (1)	Yes (1–49)	–^b^	only for research
		GnRHa	Unknown	Yes (>200)	Yes (1–49)	Yes (>200)	Yes (1–49)	Yes (not standard)	Yes (1–49)	Yes (6)	Yes (1–49)	–^b^	No
FP for male	Children (0–14 y.o)	SC	Yes (4, 5)	No	Yes (1–49)	Yes (>200)	Yes (1–49)	Yes (rare)	Yes (1–49)	Yes (6)	Yes (1–49)	No	No
		TTC	Yes (1)	Yes (1–49)	No	No	Yes (1–49)	Yes (rare)	No	No	Yes (1–49)	No	No
	Adolescents (≥15 y.o)	SC	Yes (>200)	Yes (1–49)	Yes (1–49)	Yes (>200)	Yes (1–49)	Yes^a^ (less than 100)	Yes (1–49)	Yes (6)	Yes (1–49)	Yes (1–49)	Yes (1–49)
		TTC	Yes (50–99)	Yes (1–49)	No	Mature tetsis only	Yes (1–49)	Yes (rare)	No	No	Yes (1–49)	No	No

*FP, fertility preservation; EC, embryo cryopreservation; OC, oocyte cryopreservation; OTC, ovarian tissue cryopreservation; GnRHa, gonadotropin releasing hormone agonist; SC, sperm cryopreservation; TTC, testicular tissue cryopreservation*.

a *Based on literature (49)*.

b *Detailed number is unknown*.

*The opportunities of FP for C-A patients are limited compared with FP for adult patients, because all participants (except for Indonesia) chose “not so often” for opportunities for FP for C-A patients. Also, the numbers of institutions that can provide FP treatment for C-A patients are limited*.

### Barriers That Inhibit Promotion of FP for C-A Patients

To investigate the barriers that inhibit promotion of FP for C-A patients, multiple-choice questionnaires were prepared (Table 3). Although there was variation in ranking, 9 of 11 participants identified “b: Low recognition among medical staff” as one of the major issues. Also, “f: Information is insufficient,” “a: Low recognition in society,” and “g: There is a problem with the cooperative system with the pediatrics department” were major reasons for inhibiting the promotion of FP for C-A patients (Table 3). Three of the 11 selected “e: There is technology, but we don't know how to provide it” and “j: Economically impossible.” Only one participant from Thailand chose “k: It is not necessary because the adoption system is popular.” As other comments, participants from Australia mentioned “weakness of evidence for FP for C-A patients.” To improve the level of FP awareness, 3 of 11 participants (India, Japan, Korea) are providing opportunities for lecture presentations, oral presentations at scientific conferences, and education for parents or patients.

**Table 3:** Barriers to FP for C-A patients in Asian countries.

	**Australia**	**China**	**Hong Kong**	**India**	**Indonesia**	**Japan**	**Korea**	**Philippines**	**Taiwan**	**Thailand**	**Vietnam**
a		1		1		1	3		1	1^*^	1
b	2			4	1^*^	2	1	2	2	1^*^	2
c											4
d				3							
e			1^*^	4			4				
f		2	1^*^	4	1^*^	3	2	1			3
g		3	1^*^	2	1^*^		4		3		
h											4
i											
j	1							3		1^*^	
k										1^*^	
l											
m											
Other	3										

a *Low recognition in society*.

b *Low recognition among medical staff*.

c *Medical technology is behind*.

d *Family doctor does not agree with fertility preservation*.

e *There is technology, but we do not know how to provide it*.

f *Information is insufficient*.

g *There is a problem with the cooperative system with the pediatric department*.

h *Even the prevalence of fertility preservation for adults is still low*.

i *Prohibited/limited by law or academic society*.

j *Economically impossible*.

k *It is not necessary because the adoption system is popular*.

l *Regional disparity of medical technology is large*.

m *Religious reason*.

*Other: Evidence for pediatrics is still limited (Australia)*.

* *The participants did not specify the priority order*.

*Numbers are defined in order of critical factors as “Barrier.” According to this multiple response question, “Low recognition in society and medical staff” is a major issue. Cooperative system with pediatrics department is also a big issue. Most countries have issues related to system barriers rather than technology*.

### Framework for Providing FP Treatment for C-A Patients

To improve FP treatment for C-A patients, the kinds of specialists that provided FP for C-A patients were investigated, and 10 of 11 participants replied. In half of the countries (5 of 10), only a medical doctor could provide FP treatment for C-A patients. On the other hand, in four of five countries, nurses and/or psychologists could collaborate with the medical team in FP treatment for C-A patients. Although, patient navigators as independent position and child life specialists are not involved in FP for C-A patients, in Australia, nurses and psychologist are involved as patient navigators aiming to assist decision-making and psychological support. In addition, peer supporters including cancer survivors are not involved in FP treatment for individual cases (Table 4). However, as described below, patient consumer organization and Consumer Charter are collaborating with FP organization to develop FP in Australia (20). Also, JSFP have peer supporter group to promote FP.

**Table 4:** Framework for providing FP treatment for C-A patients in Asian countries.

		**Australia**	**China**	**India**	**Indonesia**	**Japan**	**Korea**	**Philippines**	**Taiwan**	**Thailand**	**Vietnam**
Medical doctor	Oncologist and/or reproductive medicine specialist	✓	✓	✓	✓	✓	✓	✓	✓	✓	✓
	Pediatrician (Oncologist)	✓	✓	✓	✓	✓	✓			✓	✓
	Pediatrician (Other)		✓			✓					
	Pediatric surgeon	✓	✓			✓					✓
	Hematologist	✓	✓	✓	✓		✓			✓	✓
Paramedical staff	Nurse	✓				✓	✓				✓
	Social worker	✓					✓				
	Psychologist	✓		✓		✓					✓
	Patient navigator	✓									
	Child-life specialist	✓									
Others	Peer supporter	⋆				⋆					

⋆ *Australia and Japan have organizations which are consisted peer supporters and cancer survivors. However, it is difficult to attend FP treatment for individual cases*.

*In half of the countries (5 of 10), only a medical doctor could provide FP treatment for C-A patients. On the other hand, 4 of 5 countries achieved a multidisciplinary approach*.

### Resources for Providing Information About FP for C-A Patients

All of the participants selected “Oral explanation” for informed assent, and “article” is used for informed assent as supplementary material (China, Japan, Philippines, Vietnam). To improve the quality of informed assent, Korea has animations about FP treatment, including sexual education. Only Australia has an “online or printed resource” and a “video a peer supporter has done” as “other” means (Table 5).

**Table 5:** Resources for providing information about FP for C-A patients in Asian countries.

	**Australia**	**China**	**Hong Kong**	**India**	**Indonesia**	**Japan**	**Korea**	**Philippines**	**Taiwan**	**Thailand**	**Vietnam**
Oral explanation	✓	✓	✓	✓	✓	✓	✓	✓	✓	✓	✓
Illustrated book											
Article		✓				✓		✓			✓
Anime or movie							✓				
Other	✓										

*In most countries, only “Oral explanation” is the main procedure for informed assent. “Article” is used for informed assent as supplementary material (China, Japan, Philippines, Vietnam). Korea has animation about FP treatment including sexual education. Only Australia has “online or printed resource” and “video peer supporter has done*.

## Discussion

Improvement of the survival rate following childhood cancer has led to an increased focus on the late effects of cancer treatment (3, 21) and “fertility” is a prime concern for both female and male cancer survivors (3, 22) which can result in psychological distress (23). Although Asia consists of 48 countries that have various backgrounds in terms of culture, economic status, religion, and status of medical care, FP is becoming increasingly common as medical care. In particular, countries that participate in the ASFP and have specialized organizations for FP can provide contemporary FP treatment. Indeed, Australia is one of the advanced countries in the FP area, which has already established its own registration system and partial public funding for patients receiving FP treatment. Japan is also one of advanced country which has guideline of FP treatment collaborate JSFP with JSCO (Japan Society of Clinical Oncology) (24). JSFP may start a registration system for FP treatment within 1 year to understand the present status of FP in Japan based on national survey for FP (25, 26). However, FP for C-A patients is not as common as FP for adult cancer patients (27). The present study data have shown that the numbers of hospital or institutions that can provide FP for C-A patients are much fewer than for adult patients. The reason for lower number of FP in C-A patients are multi-factorial (28).

Barriers to promoting FP treatment for C-A patients may be divided into “medical factors” and others. For female C-A patients, OC and OTC are options as FP treatments, with SC and TTC for male C-A patients. In general, the selection of FP treatments depends on the patient's pubertal status. For post-pubertal female patients, EC with OC is one of the options for FP treatment (15). Although OC has been the standard FP treatment for young or unmarried female patients since 2013 as per ASCO (5), it is uncertain whether will be acceptable OC for teenagers. In fact, reports of OC for post-pubertal female patients as FP are very few, and its status is challenging, as mentioned above. Some reports and clinical data already demonstrated that OC is a practical technology for children (17), but there are issues to be resolved before pediatric fertility preservation programs can be universally available (ovarian stimulation, transvaginal procedure, sedation) (14). Furthermore, concern about delays in therapy is one of the greatest barriers to offering OC for patients (15), especially C-A patients who often require the urgent initiation of treatment due to hematological or systemic disease. In addition, OC for pre-pubertal female patients is also challenging. Although there is a report of a pre-pubertal OC patient (29), in general, only OC as a combined procedure (oocyte retrieved from ovarian cortex which extracted OTC) is available for pre-pubertal female patients (30, 31). Based on the literature, OC as a combined procedure can be available to around 40% of under 15-year-old child patients (minimum 3.5 months) (30). However, the effectiveness of the combined procedure is still very limited (32), and it has been demonstrated that the percentage of degenerated oocytes was significantly higher in girls than in adult patients (33).

OTC is the only FP treatment for pre-pubertal females and for post-pubertal patients who are unable to delay the initiation of chemotherapy, although its status is still experimental. It has been completed in patients of all ages and has been demonstrated to be safe and effective, with a low complication rate with minimal delay (15) allowing cancer treatment to commence very soon after laparoscopic surgery for OTC (34). In promoting OTC for C-A patients, the primary disease is one of the major issues. For C-A patients, leukemia is a representative primary disease. Although there is a live birth case with leukemia who received Ovarian tissue transplantation (OTT) after treatment (35), OTT following OTC in leukemia patients is challenging and requires further investigation to avoid re-introducing minimum residual disease (MRD) (10, 36). According to the European Society for Blood and Marrow Transplantation (EBMT), both pre-pubertal and post-pubertal OTC from patients with leukemia can be considered, in view of future developments, for *in vitro* maturation and subsequent *in vitro* fertilization (37). Already, as future developments, an artificial ovary and multiple-step primordial follicle culture system has demonstrated encouraging results (38). Currently, OTC has been becoming an established treatment in some countries (10); there have already been more than 130 live birth cases (11). In general, the hospital or institution that provides OTC treatment for adult patients can perform OTC for C-A patients, because both are technically the same procedure. Indeed, based on the present study, most countries that can provide OTC for adult patients replied that “it is possible to do OTC for C-A patients.” However, there are few countries that can provide OTC for C-A patients at the same level as for adult patients (although the actual numbers of OTC cases for C-A patients are unknown), due to several child-specific barriers.

For post-pubertal male patients, SC with patient assent and parent or guardian consent is an actual established method for FP (10, 34). Although the minimum age for SC is unclear (15), the success rate of SC has been reported to be up to 64.5% for adolescents aged 11–14 years (15, 39). At least Tanner stage 3 pubertal development is needed for successful SC (15, 39, 40). In general, ejaculated sperm is collected by masturbation, but penile vibratory or electro ejaculation under general anesthesia is used for patients who cannot perform masturbation (15). Also, surgical sperm extraction called “ONCO-TESE” (TESE: Testicular sperm extraction) is one of the effective procedures for patients who show cancer-induced azoospermia with a testicular tumor or lymphoma (41–43). Based on the literature, patients who underwent “ONCO-TESE” can be started on chemotherapy the same day as sperm retrieval (43). For pre-pubertal male patients, TTC is the sole treatment for FP, even though its status is still extremely experimental (10). Until now, there have been no retrievals of mature sperm or achievement of pregnancy using this treatment (15). These current situations are congruent with the findings of the present study. In conclusion, based on present survey, almost of female child cancer patients can receive OTC (except Hong Kong and Vietnam), and female adolescent patients can receive OC in Asian countries which participate this survey, although OC is uncertain whether will be acceptable for teenagers. And all adolescent male patients can receive sperm cryopreservation, also almost child male patients can receive sperm cryopreservation according to their sexual maturation (except China, Thailand, Vietnam). However, TTC for male child and adolescent cancer patients is still uncommon procedure as described above in Asia. As a limitation, age restriction was still unclear on this survey (almost participants did not clearly state). There are some possibilities that these differences to select the procedure of FP is ascribed to the developing and economical status of country.

According to the present study, there are several factors based on “medical aspects” and “social aspects” that impede the progress of FP for C-A patients. Importantly, “How to provide FP treatment for C-A” is a major issue, more so than “medical technology” as a medical factor. When we provide FP treatment for C-A patients, there are some difficulties in explaining FP treatment and obtaining informed assent/consent from children/parents. For discussion about FP with C-A patients, “Knowledge about FP (guidelines, costs, facilities and specialist, informed assent/consent process),” “low referrals,” “low priority,” “Sense of comfort for health care professionals (they feel embarrassed to discuss FP),” “Patient factors (prognosis, cost, age, feel discomfort),” “Parent factors (contradictory opinions, feel discomfort),” and “Educational resources for patients and families” (44, 45) are issues (28). Also, “provider bias” is identified as a potential barrier. Providers feel difficulties giving information about FP to patients who have low potential for fertility and/or cure, and who have a lower socioeconomic status. Furthermore, if the hospital does not have the capability to perform experimental FP treatments, it is difficult to discuss FP with patients (15, 46). These situations are among the reasons for “low referrals.” Until now, we had only five studies about decision-making for C-A patients, and all of them were performed in Western European countries (47). Therefore, we should perform surveys in Asian countries based on the different and varied cultures, including many different religions. In addition, for investigating this survey accurately, we need to consider economic status (GDP: gross domestic product) of countries, developing status of fertility treatment (especially ART: assisted reproductive technology), cost issue, distance between centers which provide FP treatment currently. And as a social aspect, difference of sanitary system is one of important factor. In Japan, the government had stated the policy for supporting young cancer patients to promote FP in 2018. Also, leading society for cancer treatment in Australia and Japan had published the guidance for FP. To promote the FP, academic societies are established in each Asian country. These societies hold opportunities of scientific meeting and symposium for advertising, dissemination in the territory.

The present study demonstrated the variety of frameworks for FP treatment among countries and the need to implement consistent oncofertility models of care in Asian countries (28). In most countries, pediatricians and pediatric oncologist/hematologist can participate in FP, but participation of pediatric surgeons is still not common. Based on the reports investigating the safety of OTC for pediatric patients by pediatric surgeons, there are no cases of delay, and they concluded that OCT is safe procedure (18). They considered port placement according to the size of the patient's body. We strongly agree with them that collaboration with pediatric surgeons is needed for OTC. The participation of paramedical staff (multidisciplinary approach) is also vital to improve FP treatment (28). According to the present study, nursing staff, social workers, and psychologists participated in FP in a few countries. Based on the national guidelines of FP for C-A patients in Sweden, involvement of a psychologist and/or counselor to give information about FP is recommended as part of a multidisciplinary approach (48). Not only medical staffs, but peer supporter and cancer survivor are important for developing FP treatment. In Australia, The FUTuRE Fertility Research Group led a collaborative consultation process with the Australasian Oncofertility Consumer group and oncofertility specialists to explore consumers' experiences of oncofertility care (20). The importance of resources (brochures and videos) for decision-making has also been emphasized (48). Although only Australia and Korea can provide video information about FP for C-A patients, most countries provide information by oral explanations. Unfortunately, there are no Asian countries in which child-life specialists and patient navigators can participate in FP treatment, likely because there are still very few child-life specialists and patient navigators in Asian countries. On the other hand, some child-life specialists are already participating in FP in the USA. As a future task, establishment of system to follow-up the reproductive issue of C-A patients after cancer treatment. In almost of Asian countries don't have system and network to follow-up C-A patients focused on reproductive issues, although some countries have guideline of long-term follow-up C-A patients.

As limitations, we investigated current status of FP for C-A patients in Asian countries, however it is difficult to compare them simply. Because they have various backgrounds of priority, culture, religion, and economical situation among them. Also, our survey had covered mainly developed countries in Asia. To assess the current status more accurately, we need to investigate remaining 34 of Asian countries which didn't participate this study.

## Conclusion

The present study demonstrated the developing status of FP for C-A patients in Asian countries. The problem that needs to be resolved is how to establish a system providing FP for C-A patients while being part of the research strategy to improve the current FP options. Asian countries hold a high value on family and so it is important that we develop an oncofertility model of care which will support the implementation of local, national and international guidelines and include healthcare providers and patients. In addition, greater consideration and more discussion needs to occur about “How to apply FP to our own society” are needed based on the various cultures and religions in the region.

## Data Availability Statement

The datasets generated for this study are available on request to the corresponding author.

## Author Contributions

ST drafted the manuscript. NSuz and AA revised manuscript. ST and NSuz designed the research and contributed to the critical discussion. JL, NM, BW, NSuk, VN, AA, DG, C-RT, AD, CL, WL, WD, R-CC, and SK contributed to collecting and analyzing data.

### Conflict of Interest

The authors declare that the research was conducted in the absence of any commercial or financial relationships that could be construed as a potential conflict of interest.

## Abbreviations

FP: fertility preservation
C-A: child and adolescent
ISFP: International Society for Fertility Preservation
ASFP: Asian Society for Fertility Preservation
JSFP: Japan Society for Fertility Preservation
FPSI: Fertility Preservation Society of India
ASCO: American Society of Clinical Oncology
JSCO: Japan Society of Clinical Oncology
EC: embryo cryopreservation
OC: oocyte cryopreservation
OTC: ovarian tissue cryopreservation
OTT: ovarian tissue transplantation
GnRHa: gonadotropin releasing hormone agonist
SC: sperm cryopreservation
TTC: testicular tissue cryopreservation
GDP: gross domestic product
ART: assisted reproductive technology.
