# Corrigendum: Fertility Preservation for Child and Adolescent Cancer Patients in Asian Countries

**DOI:** 10.3389/fendo.2020.00241

**Published:** 2020-04-28

**Authors:** Seido Takae, Jung Ryeol Lee, Nalini Mahajan, Budi Wiweko, Nares Sukcharoen, Virgilio Novero, Antoinette Catherine Anazodo, Debra Gook, Chii-Ruey Tzeng, Alexander Kenneth Doo, Wen Li, Chau Thi Minh Le, Wen Di, Ri-Cheng Chian, Seok Hyun Kim, Nao Suzuki

**Affiliations:** ^1^Department of Obstetrics and Gynecology, St. Marianna University School of Medicine, Kawasaki, Japan; ^2^Department of Obstetrics and Gynecology, Seoul National University Bundang Hospital, Seoul, South Korea; ^3^Ferticity Fertility Clinics, New Delhi, India; ^4^Division of Reproductive Endocrionolgy and Infertility, Department of Obstetrics and Gynecology, Faculty of Medicine, Universitas Indonesia, Depok, Indonesia; ^5^Department of Obstetrics and Gynecology, Faculty of Medicine, Chulalongkorn University, Bankok, Thailand; ^6^St. Luke's Medical Center, Quezon City, Philippines; ^7^Section of Endocrinology and Infertility, Department of Obstetrics and Gynecology, University of Philippines Manila, Manila, Philippines; ^8^Kids Cancer Centre and Sydney Youth Cancer Service, Sydney Children's Hospital, Randwick, NSW, Australia; ^9^Nelune Comprehensive Cancer Centre, Prince of Wales Hospital, Randwick, NSW, Australia; ^10^School of Women's and Children's Health, University of New South Wales, Kensington, NSW, Australia; ^11^Melbourne IVF, Melbourne, VIC, Australia; ^12^Division of Infertility, Department of Obstetrics and Gynecology, Taipei Medical University Hospital, Taipei, Taiwan; ^13^Obstetrics and Gynecology, The Women's Clinic, Hong Kong, China; ^14^Reproductive Medicine Center, Second Military Medical University, Changzheng Hospital, Shanghai, China; ^15^Infertility Department, Tu Du Hospital, Ho Chi Minh, Vietnam; ^16^Department of Obstetrics and Gynecology, Ren Ji Hospital, School of Medicine, Shanghai Jiao Tong University, Shanghai, China; ^17^Center for Reproductive Medicine, Tenth People's Hospital of Tongji University, Shanghai, China; ^18^Department of Obstetrics and Gynecology, Seoul National University College of Medicine, Seoul, South Korea

**Keywords:** fertility preservation, child cancer patients, ovarian tissue cryopreservation, oncofertility, Asia

In the original article, the affiliations of all, but the corresponding author, were missing. These have now been added.

The authors apologize for this error and state that this does not change the scientific conclusions of the article in any way. The original article has been updated.

